# P2X_7_ receptor inhibition attenuated sympathetic nerve sprouting after myocardial infarction *via* the NLRP3/IL‐1β pathway

**DOI:** 10.1111/jcmm.13185

**Published:** 2017-05-04

**Authors:** Jie Yin, Yu Wang, Hesheng Hu, Xiaolu Li, Mei Xue, Wenjuan Cheng, Ye Wang, Xinran Li, Na Yang, Yugen Shi, Suhua Yan

**Affiliations:** ^1^ Department of Cardiology Shandong Provincial Qianfoshan Hospital Shandong University Jinan China

**Keywords:** myocardial infarction, P2X7 receptor, sympathetic sprouting, interleukin‐1β, NLRP3, macrophage

## Abstract

Mounting evidence supports the hypothesis that inflammation modulates sympathetic sprouting after myocardial infarction (MI). The myeloid P2X_7_ signal has been shown to activate the nucleotide‐binding and oligomerization domain‐like receptor family pyrin domain‐containing 3 (NLRP3) inflammasome, a master regulator of inflammation. We investigated whether P2X_7_ signal participated in the pathogenesis of sympathetic reinnervation after MI, and whether NLRP3/interleukin‐1β (IL‐1β) axis is involved in the process. We explored the relationship between P2X_7_ receptor (P2X_7_R) and IL‐1β in the heart tissue of lipopolysaccharide (LPS)‐primed naive rats. 3′‐O‐(4‐benzoyl) benzoyl adenosine 5′‐triphosphate (BzATP), a P2X_7_R agonist, induced caspase‐1 activation and mature IL‐1β release, which was further neutralized by a NLRP3 inhibitor (16673‐34‐0). MI was induced by coronary artery ligation. Following infarction, a marked increase in P2X_7_R was localized within infiltrated macrophages and observed in parallel with an up‐regulation of NLRP3 inflammasome levels and the release of IL‐1β in the left ventricle. The administration of A‐740003 (a P2X_7_R antagonist) significantly prevented the NLRP3/IL‐1β increase. A‐740003 and/or Anakinra (an IL‐1 receptor antagonist) significantly reduced macrophage infiltration as well as macrophage‐based IL‐1β and NGF (nerve growth factor) production and eventually blunted sympathetic hyperinnervation, as assessed by the immunofluorescence of tyrosine hydroxylase (TH) and growth‐associated protein 43 (GAP 43). Moreover, the use of Anakinra partly attenuated sympathetic sprouting. This indicated that the effect of P2X_7_ on neural remodelling was mediated at least partially by IL‐1β. The arrhythmia score of programmed electric stimulation was in accordance with the degree of sympathetic hyperinnervation. *In vitro* studies showed that BzATP up‐regulated secretion of nerve growth factor (NGF) in M1 macrophages *via* IL‐1β. Together, these data indicate that P2X_7_R contributes to neural and cardiac remodelling, at least partly mediated by NLRP3/IL‐1β axis. Therapeutic interventions targeting P2X_7_ signal may be a novel approach to ameliorate arrhythmia following MI.

## Introduction

Following myocardial infarction (MI), patients are at an increased risk for ventricular tachyarrhythmia and sudden cardiac death (SCD) 1, 2, 3, while increased sympathetic nerve density is responsible for the occurrence of lethal arrhythmias 4. The mechanisms of neural remodelling have been studied extensively 3, 5, 6. Sympathetic blockade or vagal stimulation increases the VF threshold in animal studies 7, 8. There is increasing evidence that inflammation and its associated cytokines can play a key role in stimulating neurite growth and regeneration. We and others have confirmed that the macrophage is the central link between inflammation and sympathetic sprouting post‐MI 3, 6. However, the precise mechanism remains largely unknown.

Myocardial infarction leads to initial damage that is characterized by an intense inflammatory response associated with locally produced cytokines such as tumour necrosis factor‐α (TNF‐α), interferon‐γ (IFN‐γ) and interleukin‐1β (IL‐1β) 9. Of all the pro‐inflammatory cytokines, IL‐1β is well accepted as a pivotal cytokine in neuroinflammation. IL‐1β is produced predominantly by activated monocytes/macrophages and is reported to regulate the synthesis of nerve growth factor (NGF) in the non‐neuronal cells of rat peripheral nerves 10. The oligomerization domain‐like receptor family pyrin domain‐containing 3 (NLRP3) inflammasome (which consists of apoptosis speck‐like protein containing a caspase‐recruitment domain (ASC), NLRP3 and caspase‐1) is required to cleave the IL‐1β precursor to form the mature and secreted forms 11, 12. It is reported that the P2X_7_ receptor (P2X_7_R), rather than one of the other P2X receptors, acts as a costimulus or as a second signal for NLRP3 inflammasome formation as well as IL‐1β secretion from activated myeloid cells 13, 14, 15.

P2X_7_ receptor is a highly unusual ATP‐gated, non‐selective cation channel expressed primarily on cells of haematopoietic origin such as macrophages and microglia 16, 17. It is activated by high extracellular ATP levels that are released into the extracellular medium due to cell damage, hypoxia or mechanical stress, thus alerting the immune system to sites of cell damage/injury and triggering the production of reactive oxygen species (ROS) and the activation of nuclear factor kappa B (NF‐κB). In experimental AMI (Acute Myocardial Infarction), P2X_7_R has proven to be a powerful trigger for the activation of NLRP3, thus initiating the inflammatory response and worsening the prognosis 15, 18.

A direct role for macrophages and P2X_7_R in nerve and cardiac remodelling has not been explored. We hypothesized that P2X_7_R, an initial sensor for danger signal(s), is involved in modulating the inflammation and resulting sympathetic nerve sprouting post‐MI. We confirmed the activity of the P2X_7_R/NLRP3/IL‐1β axis in the heart of lipopolysaccharide (LPS)‐primed rats, examined whether P2X_7_R inhibition could attenuate sympathetic sprouting and decrease arrhythmic risk and investigated whether NLRP3 inflammasome‐mediated IL‐1β degradation is involved in this process.

## Materials and methods

### Experimental AMI model

Male Sprague Dawley rats (60–70 days postnatal, approximately 225 g; Vitalriver Company, Beijing, China) were studied in the experiment. All animals received humane care, and the study procedures were carried out according to approved protocols and guidelines established by the Animal Care Committee of Shandong University affiliated Qianfoshan Hospital (Protocol number: S 030). MI surgery was conducted after a 7‐day acclimatization period.

Each rat was anesthetized with 3% sodium pentobarbital (i.p, 30 mg/kg), intubated and ventilated as previously described 3. Following heart exposing by left thoracotomy, the left coronary artery was ligated routinely at 2–3 mm distance from origin place between the pulmonary artery conus and the left atrium 5. ST elevation, regional cyanosis and wall motion abnormalities were used as confirmation for infarction (see Fig. S1). Heating pad was set to 37°C to maintain body temperature. Three hours after coronary ligation, the survival rate was 88%.

### Experimental design

Three separate experiments were conducted.

#### Protocol 1

Twenty‐four of 27 surviving rats were divided into six groups (MI after 0, 0.5, 1, 3, 5 and 7 days; *n* = 4 per group). The temporal expression of NLRP3, P2X_7_R and mature IL‐1β was detected by Western blot.

#### Protocol 2

Twenty‐five rats were randomly divided into four groups: naive, LPS, LPS + BzATP and LPS + BzATP + NLRP3 inhibitor. The rats were stimulated with subclinical doses of LPS (2 mg/kg in normal saline, i.p.; Sigma‐Aldrich, St. Louis, Missouri, USA.), referred to as low‐dose LPS, to provide the priming signal for the expression of inflammasome components in the heart 19. Three hours later, benzoyl adenosine 5′‐triphosphate (BzATP) (4 mg/kg; Sigma‐Aldrich), a P2X7R agonist, administrated 20. The novel NLRP3 inhibitor 16673‐34‐0 (100 mg/kg in 0.05 ml; Sigma‐Aldrich) was injected subcutaneously 30 min. prior to LPS administration 21. Six hours later, the hearts were collected.

#### Protocol 3

The rats were randomly assigned so that there was approximately the same number of survivors in each group. Group A: sham surgery (*n* = 15); group B: ligation surgery (*n* = 28); group C: ligation surgery + A‐740003 (*n* = 28); group D: ligation surgery + Anakinra (*n* = 30); and group E: ligation surgery + A‐740003+ Anakinra (*n* = 28). A‐740003 was administered intraperitoneally using a previously validated dose of 50 mg/kg. The drug was given daily till the day of sacrifice starting one day prior to surgery 12, 18, 22. A‐740003 (Sigma‐Aldrich) was dissolved in distilled water and 40% hydroxypropyl‐β‐cyclodextrin 12. A total of 2 mg/kg rat recombinant IL‐1 receptor (IL‐1R) antagonist (Anakinra; R&D Systems Minneapolis, Minnesota, USA.) was dissolved in 0.67 ml of 0.9% sodium chloride and given subcutaneously immediately after surgery and every day thereafter. The doses of A‐740003 and Anakinra used in this study have been shown to exert biological effects. In each treatment group, the drugs were withdrawn approximately 24 hrs before the end of the experiments to eliminate their pharmacological actions. Samples were harvested 3 and 7 days after AMI.

### Haemodynamic measurements

Haemodynamic parameters were measured after intraperitoneal injection of 3% sodium pentobarbital (30 mg/kg). We inserted a polyethylene catheter into the left external jugular vein to perform fluid administration at the beginning. A 2‐Fr microtip P‐V catheter (SPR‐869; AD Instruments, Sydney, Australia.) was inserted into the right carotid artery, and then advanced into the left ventricular (LV) to measure heart rate, LV systolic pressure, LV end‐diastolic pressure, and maximal rates of LV pressure, increase (+dP/dt) and decrease (−dP/dt) were measured. Five consecutive pressure cycles were calculated with the application of a special P‐V analysis programme (PVAN; Millar Instruments Sydney, Australia) 23. The electrophysiological tests were then performed.

### Programmed electrophysiological stimulation

Programmed electrical stimulation was applied to all the rats to compare the incidence of inducible VAs in different groups. At the beginning of the test, the rats were intubated and ventilated as previously described. After thoracotomy, a standardized stimulation protocol was performed using electrodes implanted on the epicardial surface of the right ventricular outflow tract 24. Induced arrhythmias were caused using an electric Bloom stimulator (Chengdu Electronic Machine Company, Chengdu, Sichuan Province, China.). The induction of VAs was attempted by ventricular stimulation at a basic cycle length of 150 ms (S0) with single (S1), double (S2) and triple (S3) extra stimuli delivered after eight paced beats based on established standard 25. Once sustained ventricular tachycardia was induced, the pacing protocols were interrupted. Ventricular tachyarrhythmias, consisting of ventricular tachycardia (VT) and ventricular fibrillation (VF), was identified non‐sustained when they lasted <15 beats and sustained when they lasted >15 beats. We completed the stimulation protocols within 10 min. and applied an arrhythmia scoring system to assess the severity of the induced arrhythmia. When multiple forms of arrhythmias occurred in one animal, the highest score was used 25. All of the procedures were executed and recorded using an animal biological function experiment system (LEAD‐7000; JJET, Chengdu, China).

### Tissue collection

After electrophysiological study, the animals were sacrificed by the injection of intravenous KCl. The infarcted region was visually recognized by a mottled and pale appearance 3. The myocardium extending 0.5–1.0 mm from the infarct scar was considered to represent the infarcted myocardium. The left ventricular myocardium was cut in half through the centre of the infarct region along the baso‐apical axis. In each slice, the cardiac tissue was divided into four distinct areas that were frozen and analysed separately: (*i*) the left ventricle (LV) free wall (LVFW; >2 cm away from the infarct site, *i.e*. the remote zone), (*ii*) the right ventricle (RV) wall, (*iii*) the infarcted border region (a 3 mm zone adjacent to the infarcted area) and (*iv*) the central zone of the infarcted area. One half was immediately stored at −80°C for further biochemical analysis; the other half was fixed in 10% formalin or embedded in OCT for histological test.

### Bone marrow isolation and culture

Macrophages were differentiated from hematopoietic stem cells from the femurs and tibias of rats as described 3 and were cultured in RPMI‐1640 supplemented with 10% FBS, 100 units/ml penicillin, 100 μg/ml streptomycin and 2 mM glutamine overnight on petri dishes. The non‐adherent cells collected by centrifugation were cultured at about 1.0 × 10^6^ cells/ml with the supplement of 20 ng/ml macrophage colony‐stimulating factor (rat recombinant M‐CSF, 50 ng/ml; Sigma‐Aldrich) to harvest M‐CSF‐dependent, bone marrow‐derived macrophages (BMM). After 3 days of culture, fresh media was added. The cultured macrophages were seeded in six‐well plates at a density of (0.5–1) × 10^6^ cells/ml and incubated for 12 hrs. Cultured macrophages were randomly stimulated with LPS (10 μg/ml) and IFN‐γ (20 ng/ml), or with recombinant rat IL‐4 (10 ng/ml) for 12 hrs. Then, the stimulated macrophages were randomly divided into three groups each: sham, BzATP and Anakinra + BzATP. Anakinra (1 μg/ml) was dissolved in PBS and administered to the Anakinra + BzATP group after the cells were stimulated with LPS or IL‐4 for 2.5 hrs. After 30 min., BzATP (100 μM) was administered to the BzATP and Anakinra + BzATP groups following stimulation for another 30 min. The supernatants were collected for cytokine and growth factor analysis by ELISA, and the cells were harvested for biochemistry or immunofluorescence analysis.

### Western blotting

For WB analyses, a modified RIPA buffer (Beyotime Institute of Biotechnology, Jiangsu, China) was applied to extract total protein from frozen heart tissues of corresponding regions homogenized using a polytron blender and isolated macrophages 26. The extracted proteins from the tissues and cells were measured using the BCA protein assay reagent kit (Pierce Protein Biology St. Louis, Missouri, USA). An equal amount of total protein (80 μg of protein/lane) then resolved with a 5–12% SDS‐PAGE gel and electrotransferred onto a polyvinylidenedifluoride (PVDF) membrane (Bio‐Rad, Richmond, VA, USA). The membranes were blocked with 5% non‐fat dry milk in PBST (containing 0.05% Tween‐20) and incubated overnight at 4°C with a primary antibody against P2X_7_R (1:1500; Abcam, Cambridge, UK) or NGF (1:1000; Santa Cruz Biotechnology San Francisco, California, USA). NLRP3 (1:500; Life Span BioSciences Seattle, Washington, USA), caspase‐1 (1:500; Abcam Ltd.) and IL‐1β (1:1000, Cell Signalling Technology Danvers, MA, USA.) were measured in the clarified homogenates of tissues and cells. Following an incubation with an HRP‐conjugated anti‐rabbit secondary antibody (1:5000; ZSJQ‐BIO; Beijing, China) or an antimouse secondary antibody (1:5000; ZSJQ‐BIO) at room temperature for 1.5 hrs, the blots were developed using an enhanced chemiluminescence (ECL) detection kit (Millipore, Billerica, MA, USA) and visualized using a FluroChem E Imager (Protein‐Simple; Santa Clara, CA, USA). The bands on the blots were quantified using the NIH imageJ software (National Institutes of Health, Bethesda, Maryland, USA).

### Immunohistochemistry

Heart tissues from each group were harvested and fixed in 10% buffered formalin and embedded in paraffin for regular immunohistochemistry staining. Infarct size was evaluated on heart tissue sections stained with Masson's trichrome (Jiancheng, China) according to standard protocols 27. Only rats with a infarct size between 30% and 50% were enrolled in the study with respect to clinical importance 28. The digitized pictures were analysed by planimetry, and the infarcted area was expressed as the percentage of stained fibrosis area over total LV. Anti‐CD68 (1:150; Abcam) and anti‐P2X_7_R (1:50; Abcam) were used as the primary antibodies. The slides were incubated with an ABC Elite kit (Vector Laboratories, Burlingame, CA, USA.) and DAB substrate (Vector Laboratories) and then counterstained with haematoxylin 5.

For the immunofluorescence analysis, hearts were harvested at 7 days post‐MI and immersed in 30% sucrose diluted with PBS overnight, then embedded in Tissue‐Tek^®^ OCT compound (Sakura Finetek, Tokyo, Japan) and frozen in an isopentane liquid bath on dry ice for 10 min. before further store in −80°C. The tissues from infarcted border were incubated with anti‐TH (1:400; Millipore) and anti‐GAP 43 (a marker of nerve sprouting, 1:200; Abcam) antibodies overnight at 4°C to investigate the spatial distribution and to quantify the sympathetic nerve fibres. This process was followed by a 2‐hrs incubation with FITC‐conjugated rabbit anti‐sheep (1:200; Bethyl Montgomery, Texas, USA.) and Alexa 545‐conjugated goat anti‐rabbit (1:100; Pepro Rocky Hill, NJ, USA) secondary antibodies. We analysed four sections adjacent to the Masson's trichrome‐strained set to evaluate sympathetic innervation density; the first section taken for the analysis was 1.5–2 mm apical to the ligation 29. The density of nerve is expressed as the ratio of labelled nerve fibre area to total area (m^2^/mm^2^), while papillary muscles were excluded from the study because a variable sympathetic innervation has been reported. The CD68‐positive cells (1: 150; Abcam) in each group were double‐immunostained with P2X_7_R (1:100; Abcam) or NGF (1:50; Abcam). The sections were counterstained with DAPI (Life Technologies Billerica, MA, USA.) to identify the nuclei. For the *in vitro* study, P2X_7_R was costained with anti‐CD86 (1:150; Abcam) and anti‐CD163 (1:100; Santa Cruz). The contribution of macrophages to NGF or P2X_7_R expression in polarized macrophages was measured semi‐quantitatively by the proportion of colocalization cells (*i.e*. yellow staining in merged images) divided by the number of corresponding staining macrophages.

A total of ten microscopic fields were randomly selected, and the density was expressed as the average m^2^/mm^2^. All of the images were acquired using an Olympus LCX100 Imaging System and analysed using ImageJ software (version 1.38x; National Institutes of Health).

### Enzyme‐Linked Immunosorbent Assay (ELISA)

A double‐antibody sandwich ELISA kit (Cusabio Biotech Co, Wuhan, China) was used to detect the concentration of NGF in the collected supernatants. All of the procedures were performed according to the manufacturer's instructions. The detection range is 1.56–100 pg/ml. Each treatment was repeated three times. The mean values of NGF in the different groups were statistically compared.

### Statistics

The data are presented as the means ± standard deviations (S.D.s). Unpaired *t*‐tests were used to compare the values between two groups. anova followed by Tukey's test was used to compare the differences between more than two groups. The analyses were performed using SPSS 17.0 software (SPSS Inc., Chicago, IL, USA). A *P*‐value <0.05 was considered statistically significant.

## Results

### P2X_7_R, NLRP3 and IL‐1β protein expression is increased in rats post‐MI

**Figure 1:**
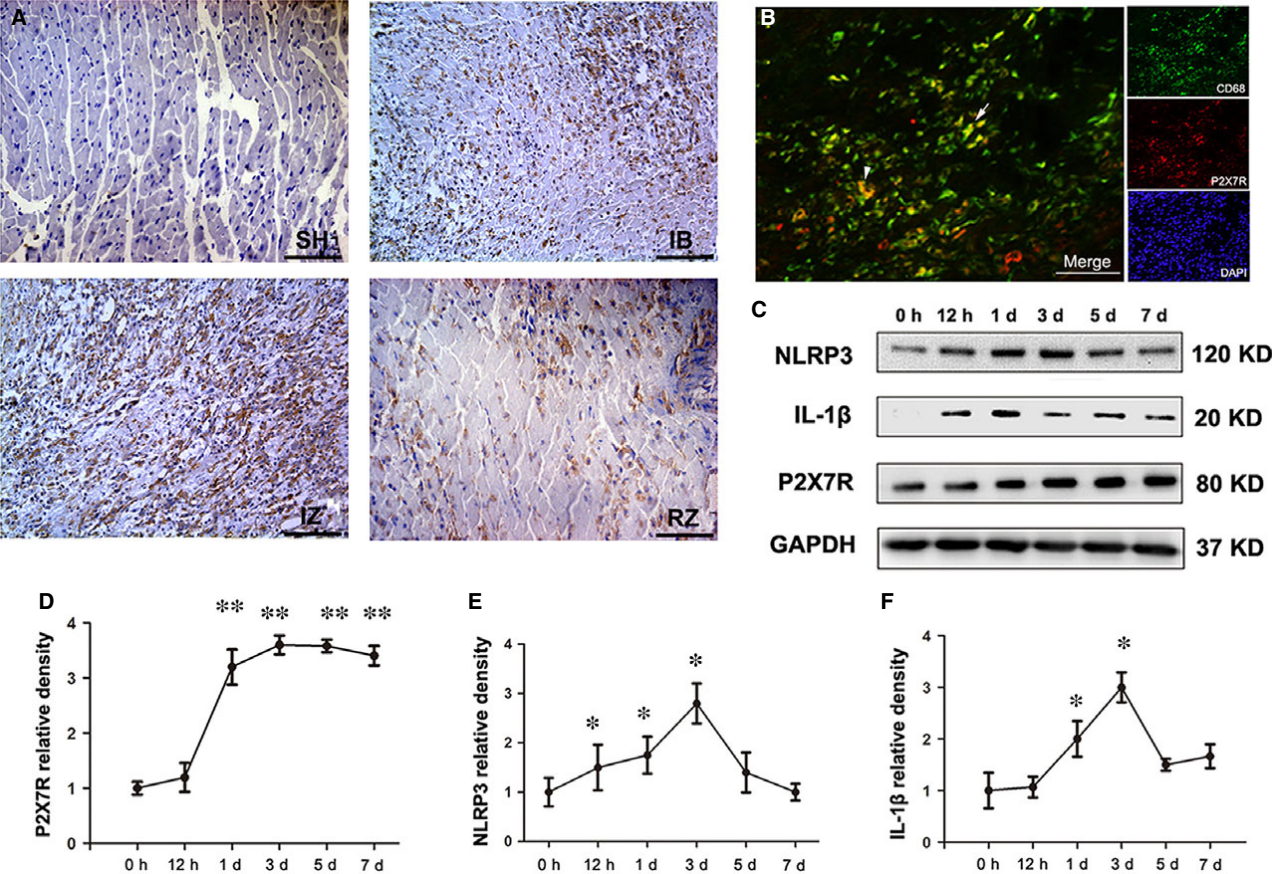
Expression profile of the P2X_7_R/NLRP3 inflammasome components after myocardial infarction (MI). (**A**) Immunohistochemical staining of P2X_7_R 3 days post‐MI in sham‐operated tissue (SH), the MI‐infarcted border (IB), the MI‐infarcted zone (IZ) and the remote zone (RZ). An increased expression of P2X_7_R was observed in the inflammatory cells and myocytes of infarcted tissue (IZ) and in the infarcted border (IB) at d3 post‐MI. (**B**) Double‐immunostaining for CD68 (green) and P2X_7_R (red) in the vehicle‐MI group showed a limited distribution of P2X_7_R on macrophages in the infarcted border. (**C**) Western blot showing the expression profiles of NLRP3 (120 kD), mature IL‐1β (17 kD) and P2X_7_R (80 kD) in the left ventricle 0 hr, 12 hrs, 1, 3, 5 and 7 days post‐MI. P2X_7_R, NLRP3 and mature IL‐1β were quantified relative to the GAPDH (37 kD) levels (**D**,** E** and **F**) (*n* = 5 per group and per time‐point). Bar = 30 μm. *P < 0.05 and **P < 0.01 *versus* 0 hr.

Immunohistochemistry implied that the non‐cardiomyocyte inflammatory cell fraction had by far the highest P2X_7_R expression in infarcted tissue and infarcted border, which was weakly expressed in cardiomyocytes and the interstitial cells (Fig. 1A). Double‐staining of CD68‐positive macrophages and P2X_7_R in the infarcted border further confirmed that nearly all of the receptors were expressed on macrophages at the early inflammatory stage (Fig. 1B). P2X_7_R was slightly up‐regulated as early as 12 hrs (although the difference was not statistically significant), significantly overexpressed at 1 day and remained at a high level after 7 days (Fig. 1D). Increased NLRP3 protein synthesis was also detected 12 hrs after surgery, reaching a peak between 3 and 5 day, and persisting up to 7 days (Fig. 1E). Parallel increases in the expression of IL‐1β were noted over a similar time period after AMI (Fig. 1F).

### LPS induces IL‐1β release in the heart *via* an ATP‐NLRP3‐dependent pathway

**Figure 2:**
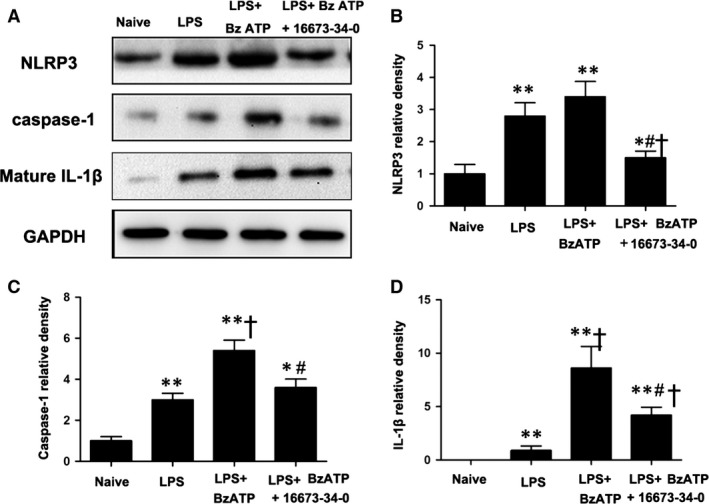
A NLRP3 inhibitor reduced inflammation induced by benzoyl adenosine 5′‐triphosphate (BzATP) in the hearts of lipopolysaccharide (LPS)‐primed rats. (**A**) Representative Western blot showing that the NLRP3 inhibitor abolished the augmentation of cleaved caspase‐1 (P20) and mature IL‐1β induced by BzATP in the LPS‐primed model. LPS alone significantly increased NLRP3 (120 kD) levels but failed to induce the production of cleaved caspase‐1 (20 kD) and mature IL‐1β (17 kD. BzATP enabled a large increase in the expression of cleaved caspase‐1 and mature IL‐1β upon LPS priming, and the NLRP3 inhibitor abolished the pro‐inflammatory response of BzATP (**B**–**D**); *n* = 6 rats per group. Each column with a bar represents the mean ± S.D. *P < 0.05 *versus* naive; **P < 0.01 *versus* naive; ^†^ *P* < 0.05 *versus* LPS; and ^#^ *P* < 0.05 *versus* LPS + BzATP.

Next, we explored whether IL‐1β generation depended on the P2X_7_‐NLRP3 signalling pathway in heart tissue. We observed that LPS priming alone significantly increased NLRP3 levels in naïve rats (*P* < 0.05, Fig. 2B) but failed to induce a robust production of cleaved caspase‐1 and mature IL‐1β. BzATP, a P2X_7_R agonist, caused the assembly of inflammasome and increased the levels of cleaved caspase‐1 and mature IL‐1β upon LPS priming (*P* < 0.01, Fig. 2C and D). NLRP3 inhibition prevented the pro‐inflammatory effect of BzATP (*P* < 0.05, Fig. 2).

### The systemic blockade of P2X_7_R inhibits the NLRP3/IL‐1β pathway and improves cardiac function

**Figure 3:**
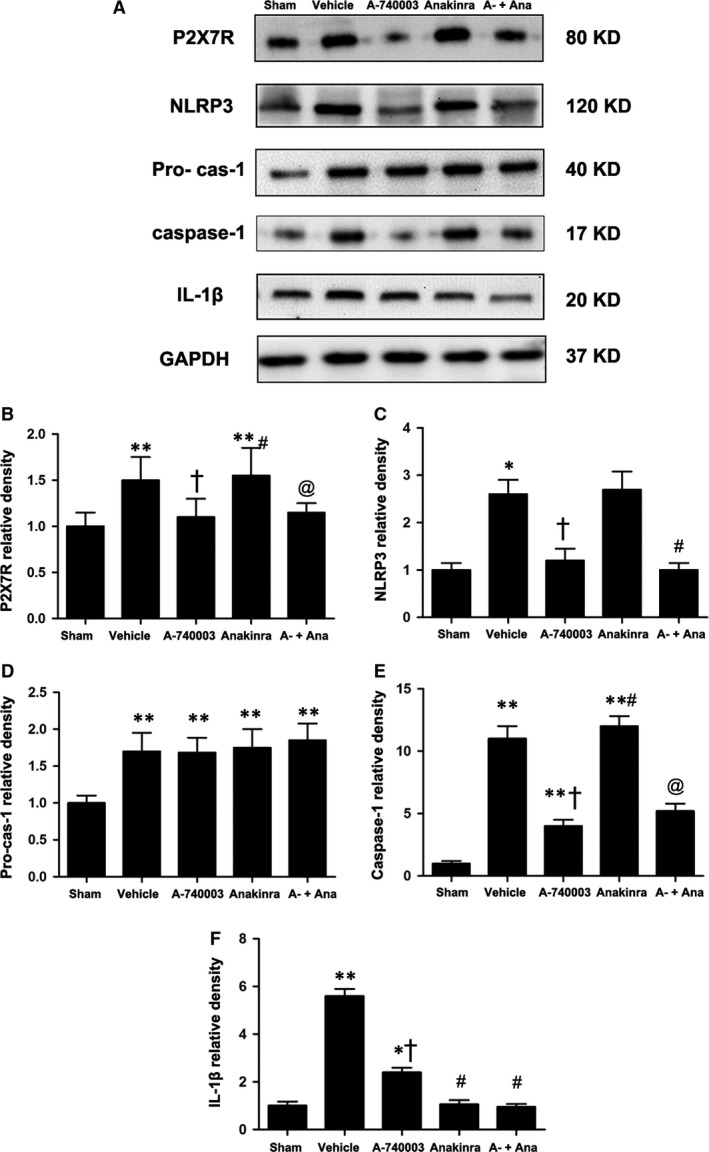
(**A**) Representative expression of P2X_7_R (80 kD), procaspase‐1 (45 kD), caspase‐1 (20 kD) and IL‐1β (17 kD) in the sham, vehicle‐MI, A‐740003‐MI, Anakinra‐MI, and A‐740003 + Anakinra‐MI groups at 3 days post‐MI as determined by Western blotting. Protein (**B**–**F**) expression was quantified relative to the GAPDH (37 kD) levels. Each column with a bar represents the mean ± S.D. **P < 0.01 and *P < 0.05 compared with sham; ^†^ *P* < 0.05 compared with vehicle; ^#^ *P* < 0.05 compared with A‐740003; and @*P* < 0.05 compared with Anakinra.

We applied the novel P2X_7_R inhibitor A‐740003 and the IL‐1R inhibitor Anakinra to explore the role of P2X_7_R and IL‐1β in the pathogenesis of MI as described in Protocol 3 16, 30, 31, 32, 33. In result, 95 of the 101 rats were enrolled (seven were excluded due to infarct area limitation and 28 died). Infarction induced marked P2X_7_R activation following a significant increase in cleaved caspase‐1 and IL‐1β protein levels compared with a relatively small increase in procaspase‐1 3 days post‐MI. The administration of A‐740003 efficiently blunted P2X_7_R up‐regulation and greatly reduced the increase in caspase‐1 and IL‐1β, while the application of Anakinra decreased IL‐1β to nearing normal levels without affecting the expression of caspase‐1 in the MI rat (Fig. 3A–E).

We evaluated cardiac morphology and haemodynamics at day 7 (Table 1). Masson's trichrome staining revealed that the infarcted area was larger in the MI‐vehicle group compared to the MI‐A‐740003 group (*P* < 0.05) and was accompanied by worsening cardiac dysfunction. Our data are supported by previous studies showing the favourable outcomes of A‐740003 and/or Anakinra 18.

**Table 1:** Cardiac morphology, haemodynamics,norepinephrine concentrations at the end of the study

Parameters	Sham		Ligation		
	Control	Vehicle	A740003	Anakinra	Anakinra+A740003
No. of surviving rats	15	18	20	21	20
Bodyweight, g	357 ± 8	348 ± 12	352 ± 10	350 ± 11	354 ± 6
Heart Rate, bpm	419 ± 14	425 ± 14	422 ± 15	424 ± 12	420 ± 11
+ dp/dt, mm Hg/sec	7102 ± 265	2814 ± 190*	3191 ± 213* ^,^ †	3085 ± 232*	3357 ± 246* ^,^ @
− dp/dt, mm Hg/sec	6853 ± 256	2403 ± 210*	2777 ± 208* ^,^ †	2478 ± 237* ^,^ #	2930 ± 223* ^,^ @
EF	79.5 ± 6.8	40.6 ± 5.8	45.2 ± 6.4	42.8 ± 6.3	50.4 ± 6.2
Infarcted area	…	58.4 ± 6%	47.3 ± 5%†	50.3 ± 4%†	46.1 ± 6%†
LVESP, mmHg	106 ± 9	92 ± 10	96 ± 6	94 ± 8	97 ± 10
LVEDP, mmHg	5 ± 1.5	14 ± 4	13 ± 5	13.5 ± 5	12 ± 5
NE, mg/g protein	1.20 ± 0.28	2.34 ± 0.30*	1.43 ± 0.26†	1.78 ± 0.34* ^,^ †	1.41 ± 0.25†

Values are mean ± S.D.

*P < 0.05 compared with respective sham. ^†^ *P* < 0.05 compared with vehicle‐treated infarcted groups. ^#^ *P* < 0.05 compared with A‐74003 treated infarcted groups. ^@^ *P* < 0.05 compared with Anakinra‐treated infarcted groups.

### The effect of P2X7 signalling on macrophage recruitment and the colocalization of IL‐1β with macrophages

**Figure 4:**
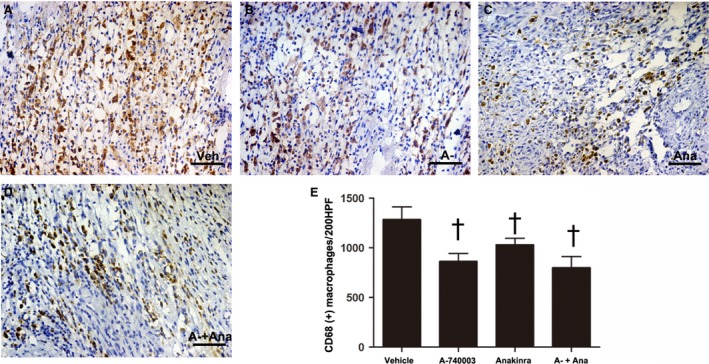
Representative immunohistochemical images of infarcted macrophage infiltration at 7 days post‐MI as indicated by the macrophage marker CD68 (yellow) and nuclei (blue) were stained with haematoxylin in the (**A**) vehicle‐MI (Veh), (**B**): A‐740003‐MI (A‐), (**C)** Anakinra‐MI (Ana) and (**D**): A‐740003 + Anakinra‐MI (A‐+Ana) groups. (**E**) Quantification of infiltrated macrophages at the infarcted border. Bar = 30 μm. ^†^ *P* < 0.05 compared with vehicle.

We measured CD68‐positive staining to quantify the degree of the inflammatory macrophage infiltration during infarction pathogenesis. CD68 expression was increased significantly after AMI and was significantly attenuated by A‐740003. On the other hand, a trend towards fewer macrophages was also observed in the Anakinra‐treated groups, although it was less than that caused by A‐740003 (Fig. 4A–D). Because macrophages are an important source of the pro‐inflammatory cytokine IL‐1β during the inflammatory phase of wound healing 34, we then double‐stained macrophages with IL‐1β to evaluate macrophage function. We found that A‐740003 and/or Anakinra significantly decreased the expression of IL‐1β in macrophages (see Fig. S2). The data indicated that P2X_7_R may be essential for the recruitment and chemotaxis of infiltrating leucocytes during AMI, which is partially mediated by IL‐1β release.

**Figure 5:**
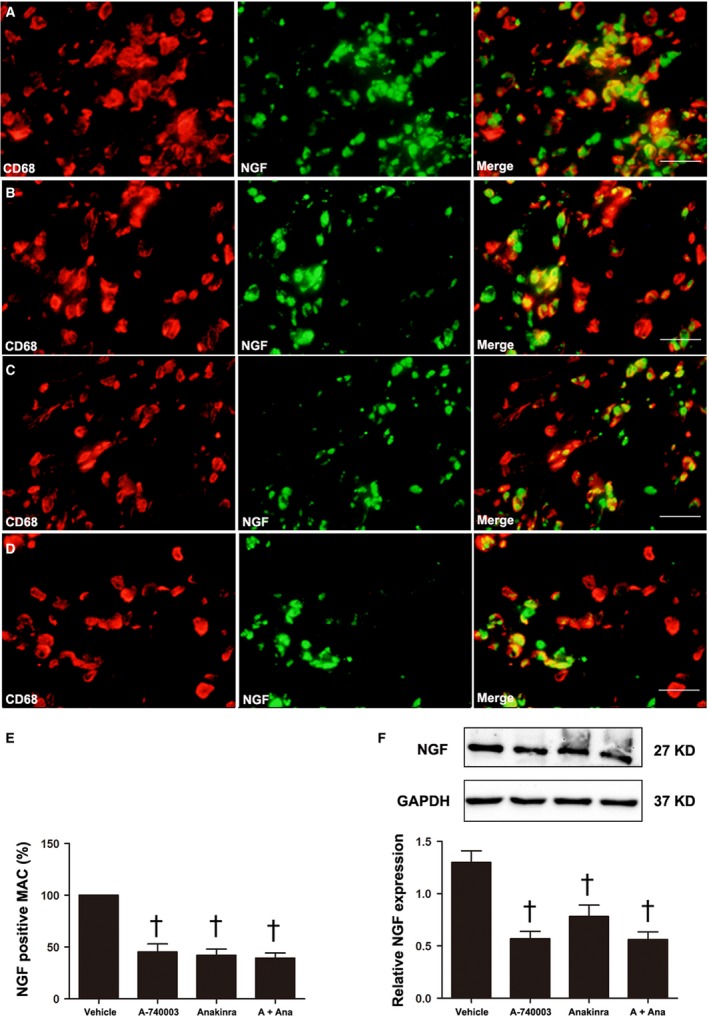
The effect of P2X_7_R and IL‐1β on macrophage‐dominated nerve growth factor (NGF) production at 7 days post‐MI. Merged images (*n* = 5) resulting from double‐immunostaining for CD68 (red) as a macrophage marker and NGF (green) in vehicle‐ (**A**), A‐740003‐ (**B**), A‐740003 + Anakinra‐ (**C**) or Anakinra‐treated rats (**D**). (**E**) The percentage of NGF‐ir macrophages. Western blot and quantitative analysis of NGF (27 kD) in homogenates of the LV from the infarcted border zone (**F**). Bar = 30 μm. The results are shown as the mean ± S.D. of three independent experiments. ^†^ *P* < 0.05 compared with vehicle.

To assess the effect of P2X_7_R inhibition on NGF, a secretory protein in the tissue, we quantified and localized NGF expression. The protein expression levels of NGF in both the infarcted border were significantly decreased, but A‐740003 was more effective at suppressing NGF than Anakinra (Fig. 5F). The NGF staining revealed that nearly all of the CD68‐positive macrophages were NGF positive (Fig. 5A–D), while in the A‐740003‐ and Anakinra‐treated rats, the proportion of NGF‐positive macrophages was decreased significantly compared with the vehicle group (*P* < 0.05) (Fig. 5E).

### P2X_7_R and IL‐1β expressed by macrophages are involved in neural remodelling in MI

**Figure 6:**
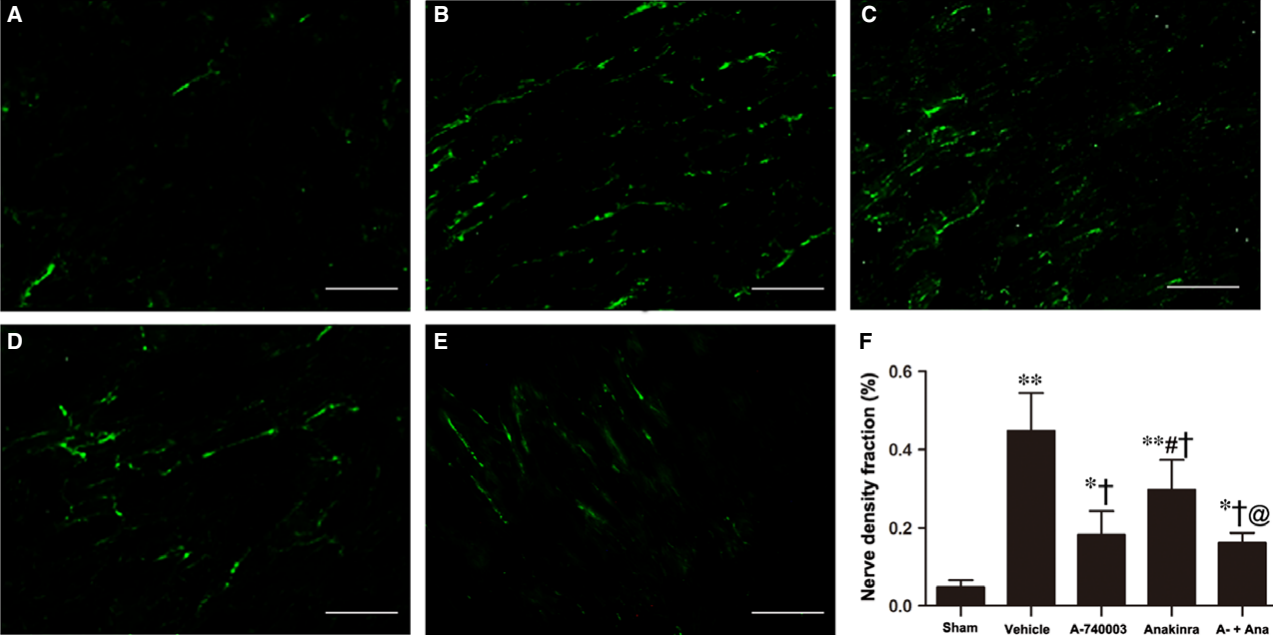
Histological study of cardiac nerve fibres at the zone in sham‐operated and infarcted hearts at 7 days post‐MI. Immunofluorescent staining for tyrosine hydroxylase (magnification 200 × ) in the (**A**) sham, (**B**) vehicle‐MI, (**C**) A‐740003‐MI, (**D**) Anakinra‐MI, and (**E**) A‐740003 + Anakinra‐MI groups. Bar = 50 μm. (**F**) Fraction of the nerve density area (%) at the infarcted border. Each column with a bar represents the mean ± S.D. **P < 0.01 and *P < 0.05 compared with sham; ^†^ *P* < 0.05 compared with vehicle; ^#^ *P* < 0.05 compared with A‐740003; and @*P* < 0.05 compared with Anakinra (*n* = 8–10 per group).

**Figure 7:**
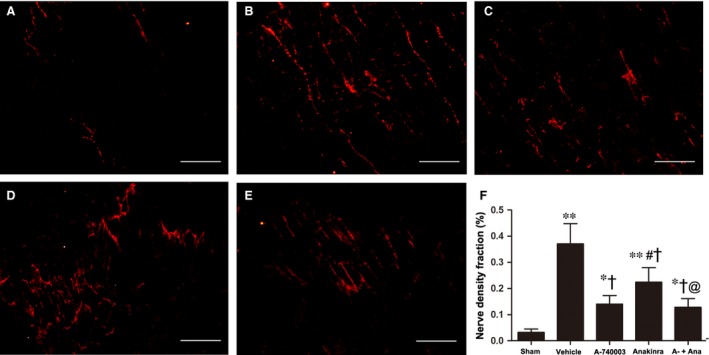
Immunofluorescent staining for growth‐associated protein 43 at the infarcted border (magnification 200 × ) in the (**A**) sham, (**B**) vehicle‐MI, (**C**) A‐740003‐MI, (**D**) Anakinra‐MI and (**E**) A‐740003 + Anakinra‐MI groups. Bar = 50 μm. (**F**) Fraction of the nerve density area (%) at the infarcted border. Each column with a bar represents the mean ± S.D. **P < 0.01 and *P < 0.05 compared with sham; ^†^ *P* < 0.05 compared with vehicle; ^#^ *P* < 0.05 compared with A‐740003; and @*P* < 0.05 compared with Anakinra (*n* = 8–10 per group).

**Figure 8:**
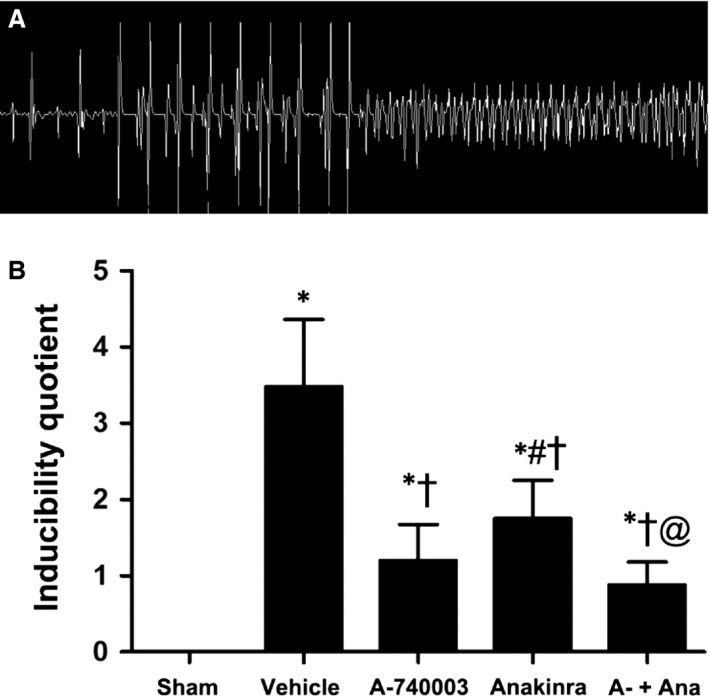
Programmed electrical stimulation at 7 days post‐MI. (**A**) Recordings of typical inducible VAs. (**B**) Comparisons of the arrhythmia scores between four groups 1 week after surgery (*n* = 5–6 for each group). *P < 0.01 compared with sham; ^†^ *P* < 0.05 compared with vehicle; ^#^ *P* < 0.05 compared with A‐740003; and @*P* < 0.05 compared with Anakinra.

The above data support the concept that the inhibition of P2X_7_R may be sufficient to blunt caspase‐1 activation and IL‐1β levels during AMI. We accessed the sympathetic nerve morphology and biological function by analysing immunofluorescent staining, cardiac norepinephrine (NE) levels and programming electrical stimulation. The distribution of TH‐immunostained nerve fibres was oriented in the longitudinal axis of adjacent myofibres (Fig. 6A–E). Significant sympathetic innervation was observed in the vehicle‐treated MI rats compared to the sham rats. To dissect the role of IL‐1β from that of the P2X_7_R/NLRP3 axis, the IL‐1R antagonist Anakinra was applied. Rats treated with A‐740003 and/or Anakinra showed significantly lower nerve density at the infarcted border zone than those treated with vehicle (0.182 ± 0.54% in the A‐740003 group, 0.298 ± 0.068% in the Anakinra group and 0.162 ± 0.022% in the A‐740003/Anakinra group *versus* 0.448 ± 0.086% in the vehicle group, *P* < 0.05; Fig. 6F), while rats treated with A‐740003 showed a lower nerve density compared with those treated with Anakinra (*P* < 0.05). No significant difference was found between the A‐740003 and A‐740003+ Anakinra groups (*P* > 0.05). Our findings demonstrating the colocalization of GAP‐43 and TH are consistent with those in other animal models of myocardial ischaemia (Fig. 7A–E). Similar to the TH results, the densities of growth‐associated protein 43, a marker for neuronal regeneration, were attenuated significantly in the A‐74003‐treated infarcted rats compared with those in vehicle‐treated infarcted rats (0.14 ± 0.30% in the A‐740003 group, 0.224 ± 0.05% in the Anakinra group and 0.123 ± 0.29% in the A‐740003+ Anakinra group *versus* 0.37 ± 0.069% in the vehicle group, *P* < 0.05; Fig. 7F). These morphometric results mirrored those of norepinephrine contents (Table 1). A significantly decreased sympathetic nerve density was also observed in parallel with IL‐1β levels (*P* < 0.05). In line with previous findings 35, lower ventricular arrhythmia also paralleled the degree of sympathetic hyperinnervation as detected by electrical stimulation (Fig. 8).

### M1 macrophages are in an essential position to produce NGF and IL‐1β

**Figure 9:**
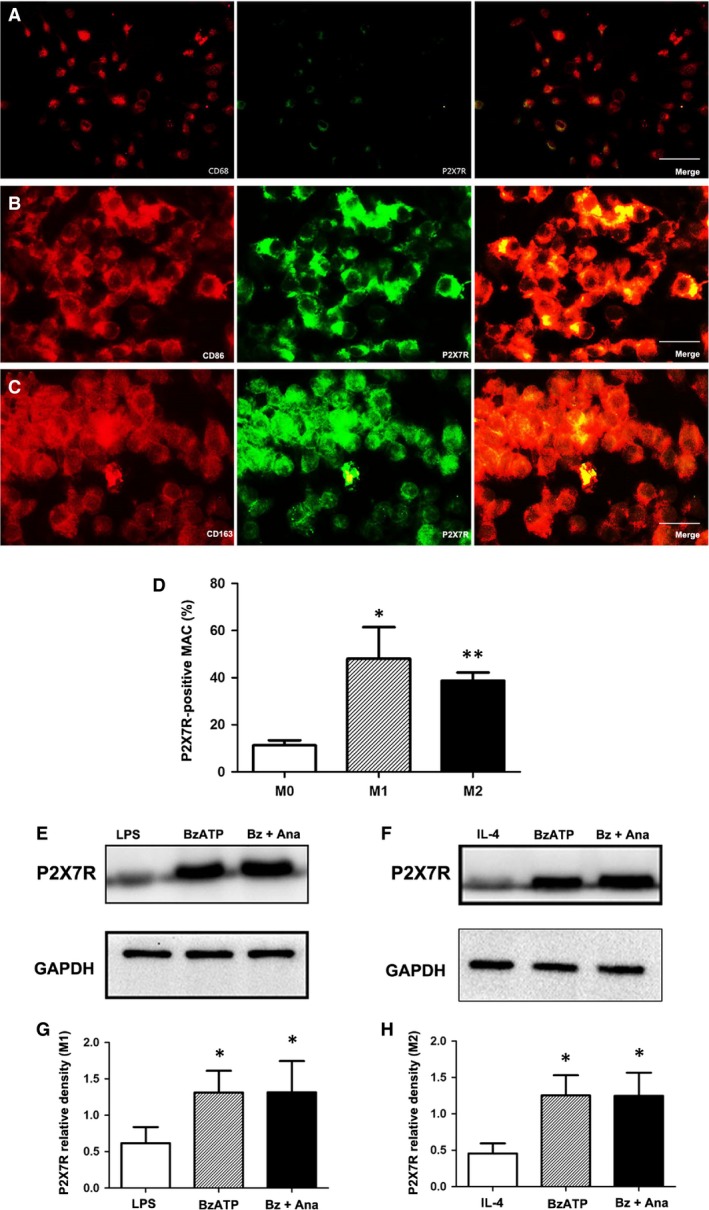
P2X_7_R expression in M1 and M2 macrophages. Representative double‐immunostained images of CD68 (**A**), CD86 (**B**) and CD163 (**C**) (red); P2X_7_R (green); and DAPI (blue) for nuclei (magnification ×400). Bar = 30 μm. (**D**) Fraction of P2X_7_R‐positive M0, M1 and M2 macrophages. Representative protein expression levels of P2X_7_R (80 kD) in M1 (**E** and **G**) and M2 (**F** and **H**) macrophages as determined by Western blotting. Protein levels were quantified relative to GAPDH (37 kD) levels. The data are expressed as the mean ± S.D. of two experiments. *P < 0.05 and **P < 0.01 *versus* the M0 (**D**) or sham groups (**G** and **H**).

Observing changes in the macrophage infiltration and functionality *in vivo* study, we aimed to characterize P2X_7_R expression and signalling *in vitro* study of isolated macrophages under the condition of stimulation. Additionally, we wanted to determine the relationship between NGF induction and P2X_7_R signalling. The LPS/IFN‐γ‐mediated bone marrow macrophages (BMM) shifting towards the M1 phenotype were characterized by the up‐regulation of CD86‐positive staining, whereas the IL‐4‐induced M2 phenotype differentiation was marked by an increase in CD163‐positive staining (Fig. 9A–C). The colocalization of P2X_7_R with both M1 and M2 macrophages was observed and further confirmed by Western blotting (Fig. 9E–H).

**Figure 10:**
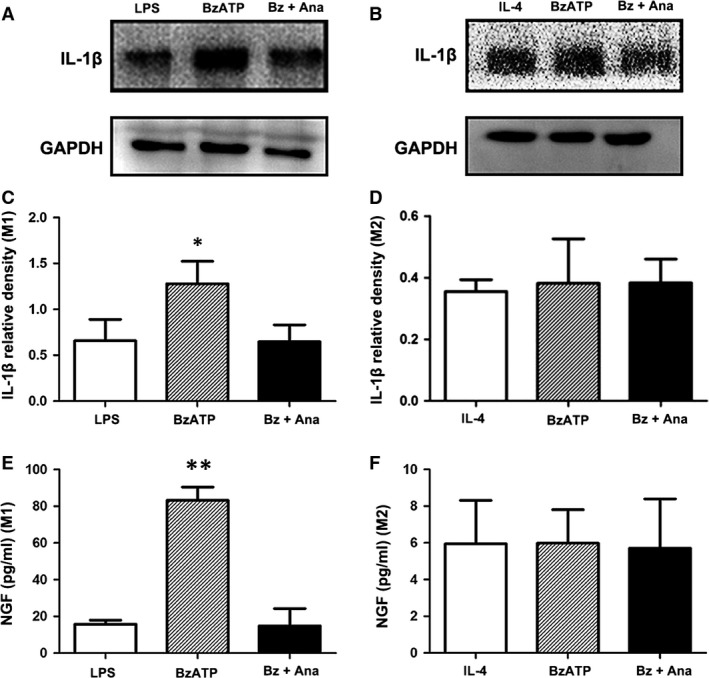
P2X_7_R activation induced nerve growth factor (NGF) release in M1 macrophages *via* IL‐1β production. Representative protein expression levels of IL‐1β (17 kD) in M1 (**A**,** C**) and M2 (**B**,** D**) macrophages in the presence or absence of Anakinra as determined by Western blotting. The quantification was carried out relative to the GAPDH (37 kD) levels. The cytokine levels of NGF in the cultured supernatants of M1 (**E**) and M2 macrophages (**F**) as measured by ELISA. Each column with a bar represents the mean ± S.D. **P < 0.01 and *P < 0.05 compared with the sham group.

Benzoyl adenosine 5′‐triphosphate could activate P2X_7_R/IL‐1β signalling in M1 macrophages (Fig. 10A); however, it produced a different effect in M2 macrophages (Fig. 10B). The activation of P2X_7_R increased the expression of IL‐1β in M1 macrophages, while the level of IL‐1β remained low in M2 macrophages, indicating that the function of P2X_7_R is mainly exerted in the M1 phenotype (Fig. 10C and D). Anakinra abolished the IL‐1β up‐regulation in BzATP‐activated M1 macrophages to a similar level observed in the sham group, while Anakinra had no impact on IL‐1β expression in M2 macrophages regardless of BzATP treatment.

We measured the concentration of NGF in culture supernatants. BzATP significantly up‐regulated NGF secretion in M1 macrophages, and this effect was further blocked by Anakinra. No obvious differences could be observed in the Anakinra + BzATP and sham groups. Furthermore, there was no difference in NGF secretion among the three groups in the M2 phenotype. The concentration of NGF in each group of M1 macrophages was generally higher than in the M2 groups (Fig. 10E and F).

## Discussion

Electrical storm (ES) describes the phenomenon of rapidly clustering ventricular tachycardia (VT) and/or ventricular fibrillation (VF) that necessitates multiple cardioversions. Most ES patients die—many within minutes or hours—especially if they have had a recent MI or ongoing myocardial ischaemia 36. The increasing sympathetic tone and sympathetic nerve density are main explanation for this, while the upstream molecular mechanism remained largely unknown. We focused on the putative role of P2X_7_R on sympathetic hyperinnervation post‐MI. The main findings in the study are as follows: (1) the P2X_7_/NLRP3 inflammasome is up‐regulated post‐MI and is significantly increased at day 3 post‐MI; (2) P2X_7_R is primarily located in non‐cardiomyocytes (*i.e*. macrophages); (3) IL‐1β secretion is meditated in a P2X_7_R/NLRP3‐dependent manner in LPS‐primed heart; (4) P2X_7_R inhibition ameliorated sympathetic sprouting *via* the NLRP3/IL‐1β pathway; (5) P2X_7_R signalling inhibition reduces inflammatory responses such as inflammatory cell infiltration and cytokine expression as well as subsequent injuries with improved cardiac function and infarct size; and (6) P2X_7_R triggers NGF secretion in M1 macrophages through IL‐1β.

Recent evidence indicates that sterile inflammation is triggered during tissue damage and that ATP is the most versatile type of danger signal 20, 37. At the onset of tissue damage, P2X_7_R is activated within seconds when cytosolic ATP flows out of ischaemic and/or necrotic cells. It is sustained at high levels in the first few days, providing a powerful secondary stimulus for NLRP3 inflammasome activation after rendering the priming PRRs active as Toll‐like receptors (TLRs). In this context, special attention is being paid to the role of P2X_7_R in the regulation of inflammatory responses following MI. Multiple pro‐inflammatory cytokines are stimulated in the pathway, with IL‐1β being the most extensively studied among them 38. In the blood samples draw from patients with coronary artery disease, the peripheral blood mononuclear cells express higher levels of NLRP3 inflammasome and the inflammasome‐dependent IL‐1β and IL‐18 cytokines when compared with the control patients 39. In animal study, NLRP3 mediates IL‐1β production post‐infarction 40, 41. In the current study, we found that P2X_7_R and NLRP3 expression in normal heart was low but increased significantly at early stages of MI. The NLRP3 is then activated in a few hours and restored at 7 days, in parallel with IL‐1β level. The peak induction at 3 days indicated a relatively early and wide therapeutic window. NLRP3 recruits ASC and procaspase‐1, the complex results in cleavage of procaspase‐1 into active caspase‐1 and IL‐1β releasing 42.

The initial signal leading to pro‐IL‐1β synthesis, an inactive cytoplasmic precursors provided through the activation of TLRs 43, must be cleaved by caspase‐1 to generate the mature active form in monocyte/macrophages 20. In consistent, we found that in the absence of acute tissue injury, priming LPS alone can elevate NLRP3 levels but is insufficient to induce inflammasome activation and IL‐1β release in the heart. In contrast, exogenous BzATP triggered NLRP3 inflammasome activation, specifically promoting the maturation and release of IL‐1β, which was further reversed by a NLRP3 inhibitor. The above data suggested that the P2X_7_R/NLRP3/IL‐1β axis is exist in heart tissue and is involved in MI. The precise mechanism of mature IL‐1β release is not clear but is known to require K^+^ efflux followed by the assembly of the NLRP3 inflammasome as K^+^ efflux agonists were shown to induce NLRP3 inflammasome activation independently of Ca^2+^ signalling 44.

Next, we applied P2X_7_R and IL‐1β inhibitor to assess the role of P2X_7_R signalling in the pathogenesis of MI. To inhibit P2X_7_R, we applied the novel A‐740003, which represents a significant advance in P2X_7_R pharmacology and is more potent, more selective and less species‐specific in various preclinical models of disease 16, 30. For IL‐1 inhibition, we used Anakinra, which has been tested in both clinical trials in prior AMI patients and in experimental models in rodents 31, 32, 33. The results of our experiments demonstrate that the inhibition of P2X_7_R by A‐740003 is sufficient to suppress the earliest steps in post‐infarction inflammatory cascades, blunt caspase‐1 activation and inhibit the increase in IL‐1β levels, suggesting that P2X_7_R participate the inflammation process post‐MI.

Inflammation following nerve injury modulates reinnervation *via* a cytokine/neurotrophin axis. Of all the cytokines, IL‐1β is mostly involved in various neuropathies such as diabetic peripheral neuropathy 45, 46, 47. Besides, most of the pharmacological evidence regarding the therapeutic potential of targeting P2X_7_R was focused on the processing and release of IL‐1β from activated monocytes/macrophages and microglia 48, 49, 50. These results led us to explore whether P2X_7_R‐mediated IL‐1β releasing participated in the process of sympathetic hyperinnervation.

**Figure 11:**
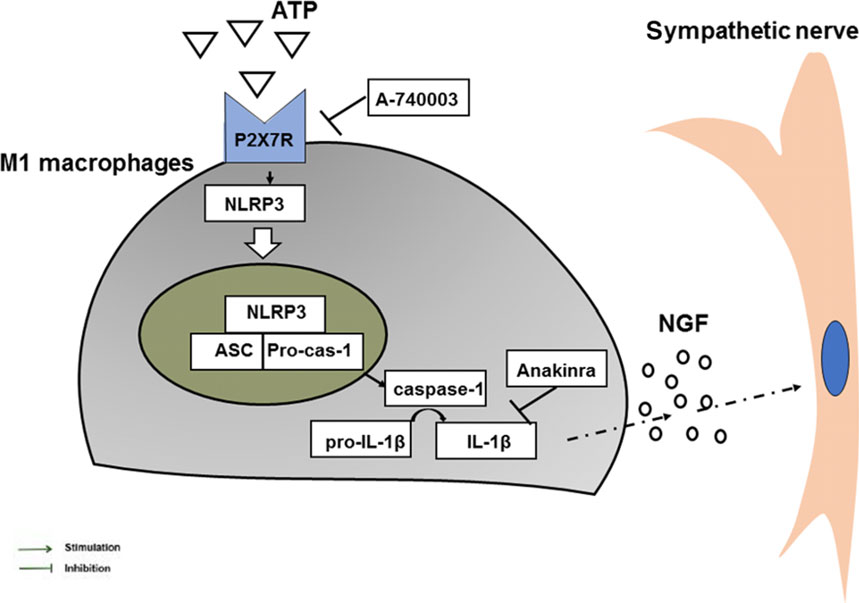
Schematic of a hypothetical mechanism for the role and mechanism of P2X_7_R in sympathetic hyperinnervation post‐MI.

We found that P2X_7_R has a restricted cellular distribution in infarcted heart, mainly colocalized with macrophages. IL‐1β is reported to be secreted mainly by macrophages and myofibroblasts instead of resident cardiomyocytes 18. Myocardial macrophage infiltration was markedly reduced by A‐740003. Hence, P2X_7_ may orchestrate macrophage‐dominated inflammation in the MI process. Macrophages are well‐recognized elements that link the immune and nervous systems through the synthesis of NGF. The injection of monocytes/macrophages into the immediate surroundings of peripheral nerve cell bodies significantly enhanced axonal regeneration 51; in contrast, macrophage depletion or regulation post‐MI markedly reduced NGF expression and presented to be a potential regulatory target for cardiac sympathetic hyperinnervation 3, 6; however, the mechanism remained unexplored. Our data showed that P2X_7_R inhibitors significantly decreased the proportion of NGF‐ir macrophages. Consistently, the density of GAP‐43‐ and TH‐positive nerve fibres was clearly decreased, and hyperinnervation was prevented in A‐740003‐treated animals. This may indirectly reduce sympathetic outgrowth by modulating NGF, although it is not known to directly elicit sympathetic sprouting 52. Moreover, the neutralization of IL‐1R reduced NGF production substantially, but still less effective than the inhibition of P2X_7_R. In accordance, the nerve density level in Anakinra‐treated rats was higher than that in A‐740003 treated rats. The reason for this could be that Anakinra is less effective at reducing macrophage infiltration than A‐740003 as macrophage is the main source of NGF during the inflammatory stage of MI. The addition of Anakinra following A‐740003 administration did not further reduce sympathetic sprouting, suggesting that the deleterious effects of MI are mediated, at least in part, by IL‐1β. Therefore, we speculated that P2X_7_R may modulate NGF‐related sympathetic hyperinnervation through IL‐1β. Previous study indicated that the secretion of IL‐1β by recruited macrophages early after infarction is important for initiating Schwann cell mitosis and nerve innervation in the setting of MI, which evidenced our speculation 4, 19. Our *in vitro* study further that showed that the expression of NGF in M1 macrophages occurs in a P2X_7_R/IL‐1β‐dependent manner, whereas the M2 subtype does not appear to be involved in that process. Taken together, these studies revealed the mechanism by which P2X_7_R regulates NGF production and sympathetic hyperinnervation under inflammatory condition (Fig. 11). Consequently, ventricular arrhythmia score was reduced following ameliorated sympathetic innervation, supported by previous study reported that sympathetic blockade or vagal stimulation reduces the VF threshold in animal studies 53.

Addition to the beneficial effect on neural remodelling, P2X_7_R inhibition showed improved cardiac function in a short observational time of 7 days, which predicted reduced development of clinical VT by permitting re‐entry distributed within the infarct border zone in a long term 54.

### Outlook

The knowledge gained about IL‐1β from clinical trials preceded the finding of the inflammasome. In addition, the effect of P2X_7_R antagonists on several inflammatory conditions has been testified by a number of clinical phase I and II trials 55. Our data provide a strategy by which to limit secondary damage after MI using the broadly available, inexpensive, well‐tolerated and systemically potent P2X_7_R inhibitor A‐740003. We found a specific role for P2X_7_R in macrophage‐dominated inflammation post‐infarction. Our future studies will mainly focus on the cell‐specific role of P2X_7_R activation in myocardial infarction injury by applying an irradiation/bone marrow transplantation (BMT) model. In addition, selective myeloid inhibitors should be developed.

### Limitations

Firstly, we should understand the potential limitations of extrapolating data from rats to humans; secondly, because the infarct size could not be measured before drug administration, structure change data could somehow be affected by the ligation; thirdly, the cellular sources of P2X_7_R and NLRP3 expression and IL‐1β production were not examined in the present study; and fourthly, although we targeted pathogenic events in multiple cell types (macrophages, neurons and fibroblasts) involved in cardiac injury, P2X_7_R inhibitors may prove superior in a single cell type. In line with previous studies, P2X_7_R/NLRP3 activation was mainly observed in myeloid cells such as macrophages. Thus, further studies are needed to specifically elucidate the macrophage contribution to neural remodelling. However, despite such limitations, the results of our study point towards a key beneficial effect of the P2X_7_ signalling pathway in post‐infarction remodelling.

## Conclusions

Collectively, these data clearly indicate that in macrophages, P2X_7_R modulates IL‐1β *via* the P2X_7_/NLRP3 pathway, and in doing so, it contributes to the inflammatory state post‐infarction. A‐740003 ameliorates neural and cardiac remodelling that is meditated at least partly by NLRP3/IL‐1β. This opens a new therapeutic opportunity for the prevention and treatment of MI.

## Conflict of interest

All authors have no conflict of interest to declare.

## Supporting information


**Fig. S1** Confirmation of myocardial infarction by Masson's trichrome staining of the infarcted area (A, B); elevated ST (top 2) and ventricular tachycardia and ventricular fibrillation.
**Fig. S2** Merged images resulting from double‐immunostaining for CD68 (red) as a macrophage marker and IL‐1β (green) in vehicle‐ (A), A‐740003‐ (B), A‐740003+ Anakinra‐ (C) or Anakinra‐treated rats (D). (E) The percentage of IL‐1β‐ir macrophages
