# Supplementary material for: P2X7 receptor inhibition attenuated sympathetic nerve sprouting after myocardial infarction via the NLRP3/IL‐1β pathway
Source: J Cell Mol Med. 2017 May 4;21(11):2695–710. doi: 10.1111/jcmm.13185 (PMC5661108; doi:10.1111/jcmm.13185)
Supplement: Supplementary file 1 — Fig. S1 Confirmation of myocardial infarction by Masson's trichrome staining of the infarcted area (A, B); elevated ST (top 2) and ventricular tachycardia and ventricular fibrillation. Fig. S2 Merged images resulting from double‐immunostaining for CD68 (red) as a macrophage marker and IL‐1β (green) in vehicle‐ (A), A‐740003‐ (B), A‐740003+ Anakinra‐ (C) or Anakinra‐treated rats (D). (E) The percentage of IL‐1β‐ir macrophages [file JCMM-21-2695-s001.docx]

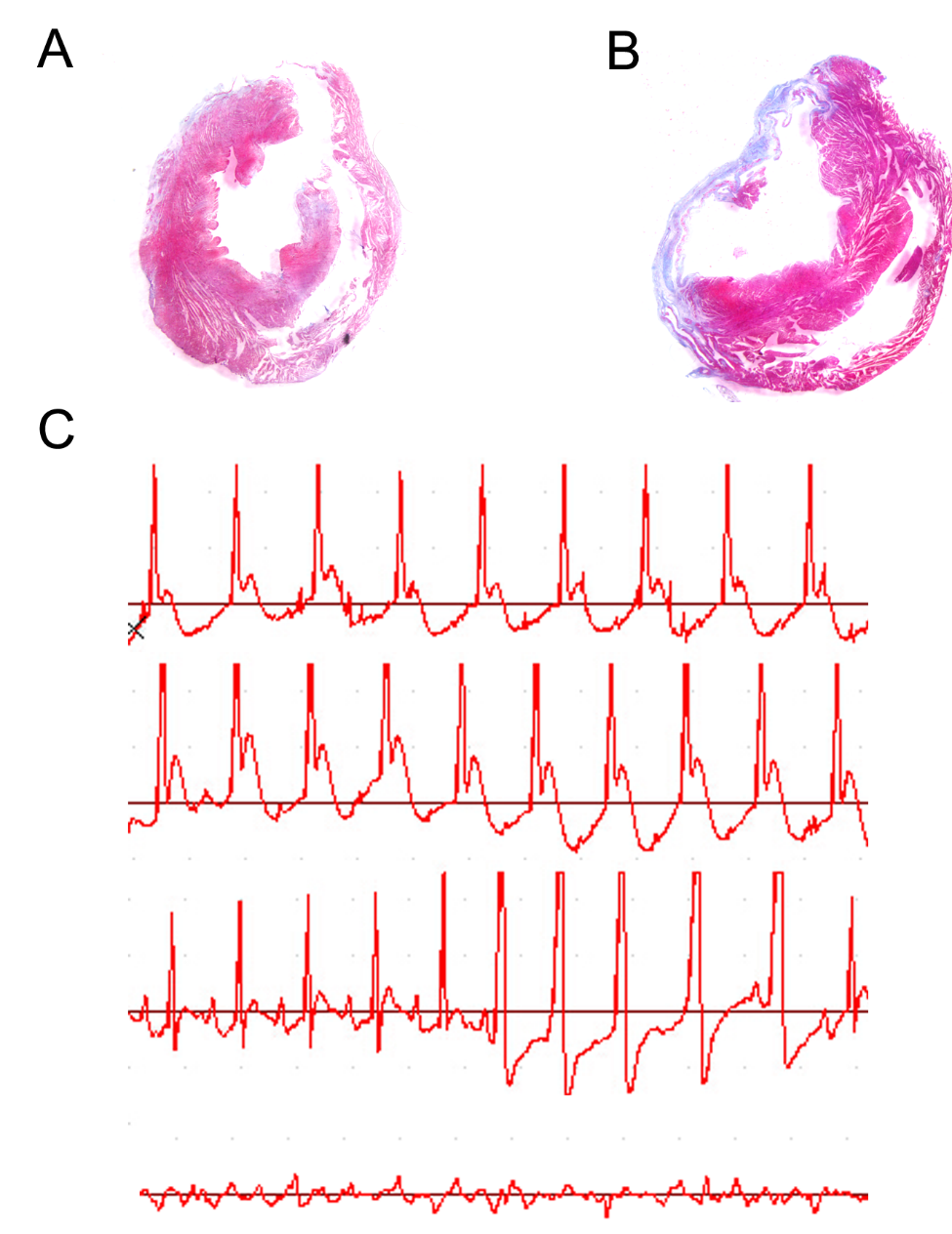


**Supporting information 1 (S1):** Confirmation of myocardial infarction by Masson’s trichrome staining of the infarcted area (A, B); elevated ST (top 2) and ventricular tachycardia and ventricular fibrillation.


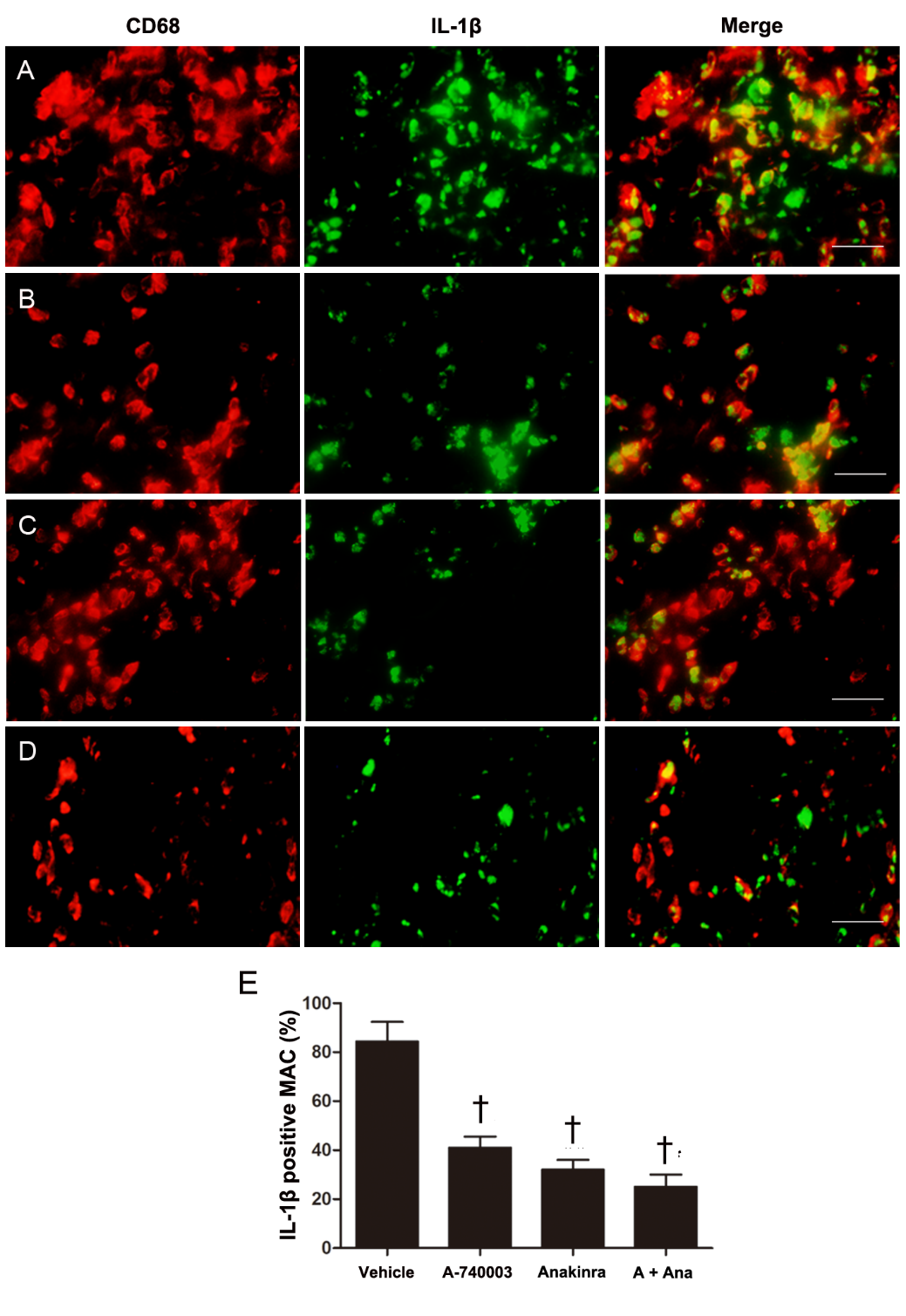


**Supporting information 2(S2):** Merged images resulting from double-immunostaining for CD68 (red) as a macrophage marker and IL-1β (green) in vehicle- (A), A-740003- (B), A-740003 + Anakinra- (C) or Anakinra-treated rats (D). (E) The percentage of IL-1β-ir macrophages. Bar = 30 μm. The results are shown as the mean ± SD of three independent experiments. †p<0.05 compared with vehicle.
